# Methods to Investigate the Secretome of Senescent Cells

**DOI:** 10.3390/mps7040052

**Published:** 2024-07-02

**Authors:** Afshin Samiminemati, Domenico Aprile, Dario Siniscalco, Giovanni Di Bernardo

**Affiliations:** 1 Department of Experimental Medicine, Biotechnology, and Molecular Biology Section, Luigi Vanvitelli Campania University, 80138 Naples, Italy; afshin.samiminemati@unicampania.it (A.S.); domenico.aprile@unicampania.it (D.A.); dario.siniscalco@unicampania.it (D.S.); 2 Sbarro Health Research Organization, Temple University, Philadelphia, PA 19122, USA

**Keywords:** senescence, secretome, SASP, spectrometry, RNA sequencing

## Abstract

The word “secretome” was first used to describe the proteins that cells secrete under different circumstances; however, recent studies have proven the existence of other molecules such as RNA and chemical compounds in the secretome. The study of secretome has significance for the diagnosis and treatment of disease as it provides insight into cellular functions, including immune responses, development, and homeostasis. By halting cell division, cellular senescence plays a role in both cancer defense and aging by secreting substances known as senescence-associated secretory phenotypes (SASP). A variety of techniques could be used to analyze the secretome: protein-based approaches like mass spectrometry and protein microarrays, nucleic acid-based methods like RNA sequencing, microarrays, and in silico prediction. Each method offers unique advantages and limitations in characterizing secreted molecules. Top-down and bottom-up strategies for thorough secretome analysis are became possible by mass spectrometry. Understanding cellular function, disease causes, and proper treatment targets is aided by these methodologies. Their approaches, benefits, and drawbacks will all be discussed in this review.

## 1. Introduction

The term secretome was introduced in 2000 in a study of secretory processes in *Bacillus subtilis*, and by Tjalsma, to refer to a variety of factors that are secreted by cells through various situations [1,2]. Accordingly, this definition was changed in 2010 from secretory constituents to released proteins [3]. Secretome, sometimes also referred to as “secretomics”, has recently emerged to describe the comprehensive analysis of proteins produced by a cell, tissue, or organism under specific conditions or at a particular point in time [4].

These secreted proteins are involved in immunological responses, development, adhesion, proteolysis, homeostasis, and extracellular matrix organization. The chemical composition of the secretome is very dynamic, changing in response to different environmental stressors and disorders. Mechanistic insights could be obtained by intracellular route and network studies, which establish connections between proteins and other crucial members participating in these processes, besides their underlying cellular activities. There are various secreted complexes from cells; nevertheless, there is still a lack of proper understanding of their size, composition, and their functions. Based on previous research, it has been suggested that the secretome of cells could be involved in signaling pathways to have different functions, for instance, protein-like growth factors, hormones, and cytokines [5,6,7]. According to the study of Sung et. al, astrocytes play a crucial role by secreting several factors, including growth factors and cytokines for the maintenance and communication of central nervous system [6]. The secretome of cells encompasses a diverse array of molecules beyond proteins [8]. It includes not only soluble proteins such as cytokines, chemokines, and growth factors but also extracellular vesicles (EVs) like exosomes and microvesicles. These EVs contain various molecular cargoes, including microRNAs (miRNAs), messenger RNAs (mRNAs), and other nucleic acids, which play significant roles in intercellular communication and modulation of physiological processes. EVs play crucial roles in intercellular communication by encapsulating cytosolic components, metalloproteins, and matrix-remodeling enzymes. Particularly in senescent cells, EVs contribute to various processes influencing tissue homeostasis, aging, and disease. For example, EVs released by senescent cells propagate senescence signals to neighboring cells, thereby spreading the senescent phenotype within tissues. Additionally, EVs facilitate communication between senescent cells and their neighbors, enabling the transfer of microRNAs, non-coding RNAs, proteins, and lipids. These molecules can modulate gene expression, alter cellular pathways related to proliferation and differentiation, and influence tissue-wide senescence dynamics [9].

Additionally, the secretome can harbor lipids and metabolites, further contributing to its functional complexity in processes such as tissue repair, immune modulation, and cellular signaling [10]. The composition of the secretome is dynamic and can vary significantly depending on the cell type, physiological state, and external stimuli, underscoring its complexity and the multifaceted nature of cellular secretions.

Furthermore, investigating the cell-secreted proteins not only might lead to creating specific treatments for a variety of diseases, but also could be beneficial to diagnose disorders in patients [11]. It has been shown that the overlapping of canonical pathways of the secretome of multilineage-differentiating stress enduring (MUSE) cells has led to identifying the biological activities that could decrease apoptosis, immunomodulation, and stem cell self-renewal capability [12,13].

The MSC secretome plays various tasks in niche and tissue regeneration since it contains cytokines, growth factors, extracellular vesicles, and other signaling molecules. Accordingly, MSC secretome could contribute to the therapeutic effects through several mechanisms, including immune response, stimulation of tissue regeneration, angiogenesis promotion, cell-to-cell communications, exertion of paracrine effects, and extracellular vesicle and exosomes. Cellular senescence could alter the MSC secretome through changing the properties of secreted factors.

### Senescent Cells Unmasked: Exploring Their Distinctive Traits and Properties

Cellular senescence, a state characterized by arrested cell division and functional loss, appears following telomere shortening, non-telomeric DNA damage, and excessive mitogenic signals [14]. To address the senescent cells’ features, permanent cell cycle arrest, production of a specific type of secretome which is called SASP, morphological changes, metabolic and functional changes, chromatin and DNA damage, telomere dysfunction, and being resistant to apoptosis could be mentioned.

This process can be categorized into early, middle, and late stages, each with distinct characteristics and functional implications [15]. Early senescence occurs when cells first encounter stress, activating tumor-suppressor pathways, notably involving p53 and retinoblastoma protein (pRb), leading to cell cycle arrest [16]. During this phase, cells initiate to establish the senescence-associated secretory phenotype (SASP), which involves the secretion of pro-inflammatory cytokines, chemokines, growth factors, and proteases.

Middle senescence represents a transitional period where the senescent state becomes more stable and irreversible. The SASP becomes more pronounced, and cells exhibit robust changes in gene expression. During this phase, cells strengthen their growth arrest mechanisms and enhance their secretory profiles, significantly influencing the tissue microenvironment. This stage is crucial for modulating the immune response and tissue remodeling, as well as in tumor suppression by limiting the proliferation of damaged cells.

Late senescence is characterized by fully established and stable senescence markers, such as the persistent expression of senescence-associated β-galactosidase (SA-β-gal). Cells in this phase exhibit extensive chromatin remodeling, known as senescence-associated heterochromatin foci (SAHF), and a sustained SASP. The secretome of late senescent cells can have both beneficial and detrimental effects; while it can reinforce the arrest of neighboring damaged cells and aid in tissue repair, its chronic secretion can contribute to inflammation, tissue dysfunction, and age-related pathologies if senescent cells accumulate and are not efficiently cleared by the immune system. Understanding these stages of cellular senescence is essential for developing therapeutic strategies aimed at mitigating the adverse effects of senescent cells in aging and age-related diseases while harnessing their beneficial roles in cancer suppression and tissue repair [17].

On the one hand, senescence acts as a natural defense against cancer by halting the proliferation of transformed cells [18]. On the other hand, it actively contributes to the aging process by releasing SASP. The SASP performs a multitude of tasks; it can sensitize nearby cells to senescence, preventing situations where adjoining cells with insignificant DNA damage endure unaffected. Bizarrely, it can also wreak havoc on tissue and organ functionality, accelerating the organism aging [19].

The SASP could have a multifaceted role in various biological processes, impacting both tissue homeostasis and pathology. Furthermore, it might attract and trigger immune cells to eliminate senescent cells [20]. To mention some other features of senescent-secreted secretome, the following characteristics could be considered: paracrine signaling which is able to propagate the senescence signal, immune surveillance, and clearance through recruiting immune cells such as macrophages, natural killer cells, and T cells to the site of senescence [20], tissue repair by promoting the proliferation and differentiation of stem cells, or tissue regeneration through enhancing angiogenesis and extracellular matrix remodeling [21]. There are studies that demonstrated the effect of senescence on tissue repair and development [21]. The ability of senescence to reduce the growth of cancer cells by blocking the proliferation of transformed cells has also been reported [22]. In the initial exposure, SASP could act as tumor suppressive by reinforcing cell cycle arrest and preventing the proliferation of oncogenic cells as well as activating immune-mediate clearance of pre-malignant cells. In the adverse way, the chronic SASP signaling can promote tumorigenic effects through creating a pro-inflammatory environment which is able to support tumor growth and progression.

It is argued that higher SASP levels might be linked to biological and chronological aging [23]. Despite the possibility to measure the distinctive features of senescent cells in isolated tissues, the application of that in human clinical treatments needs to cope with a plethora of challenges. Utilizing the circulating SASP as a measure of the overall load of senescent cells in the system may be very beneficial, as the SASP is a major pathogenic characteristic of senescent cells. This could also be useful for identifying patients who would have shown the best results against new treatments in clinical research.

Furthermore, by the study of the cell and tissue secretome, an in-depth view of human diseases and treatments could be provided which might be useful for finding potential approaches in clinical treatment studies. There are various approaches for studying the compositions of cellular secretome. This study aimed to discuss the different methods for analyzing the secretome of senescence cells.

## 2. In Silico Analysis of Cell Secretome

Thanks to the improvements in bioinformatics and in silico studies, it is now possible to gain some information about this complex without applying any wet lab activities. Nowadays, bioinformatics has a critical role in various fields of biology for prediction and having new ideas for laboratory experimental design. Particularly for secretome analysis, there are different databases, like SecretomeP and SignalP, for anticipation of secretome components, which use the information based on the signal peptide. Despite the advantages of this method, such as less time and expenses, there is the risk of false-negative and false-positive results. Also, it has been proven that the only bioinformatics prediction could not be enough as it might have some errors; at least for secretome analysis, in which those proteins lacking signal peptide could not be encountered by the software [24]. Many proteins are now known to be secreted by non-classical secretory routes [25]. This class of proteins, that are secreted without containing signal peptide, are secreted through a variety of mechanisms, such as cell surface shedding and inclusion in exosomes and secretory vesicles [26].

Previously, it was believed that secretome proteins could only be released by traditional secretion channels, which used N-terminal signal peptide signatures. The most popular tool for non-classical secretory proteins, SecretomeP [27], is often utilized to predict non-classical secretory proteins. Nevertheless, SignalP is the other tool which can predict the secreted proteins containing signal peptides which are expressed through the classical pathway [28]. The signal peptides normally have a length of 15–30 amino acids and despite having a similar structural construction, they do not exhibit strong sequence homology [29]. In this main, the previous idea was that only the proteins which have signal peptides, could be secreted. Nevertheless, recent research has demonstrated that proteins lacking signal peptides can also be secreted through non-classical pathways. These non-classical secretory mechanisms include the release of proteins through exosomes, microvesicles, or other unconventional pathways that do not involve the endoplasmic reticulum–Golgi apparatus route [30]. This discovery has expanded our understanding of protein secretion, revealing that cells can employ diverse strategies to secrete a wide array of proteins, thus playing significant roles in intercellular communication and various physiological processes.

There have been several studies into the mammalian secretome, even though the first secretome analysis was carried out in bacteria and fungi [31]. It is believed that the proportion of secretome proteins is around 10% of the 20,500 human protein-coding genes [32]. A compilation of more than 18,000 secreted proteins from the proteomes of humans, mice, and rats is listed in the Secreted Protein Database (SPD, http://www.hsls.pitt.edu/obrc/index.php?page=URL1104935692 accessed on (29 June 2024) which contains sequences from SwissProt, Trembl, Ensembl, and Refseq [33]. The MetazSecKB is the other database which considers downloadable secretome predictions for a variety of metazoan species, including a few vertebrates, was just released by Meiken et al. in 2015 [34]. In Table 1, a summary of the in silico methods and their advantages and disadvantages is shown.

## 3. Secretome Analysis Methods Based on Protein

Secretome analysis methods focusing on proteins involve directly identifying and quantifying proteins secreted by cells into the extracellular environment, offering insights into cellular communication, cellular senescence, and other conditions. Central to this approach is mass spectrometry (MS), which provides high-resolution and high-throughput profiling of secreted proteins. Techniques such as enzyme-linked immunosorbent assays (ELISAs) and Western blotting are also utilized for targeted validation and quantification of specific proteins within the secretome. Advanced methods like stable isotope labeling by amino acids in cell culture (SILAC) enable quantitative proteomics, allowing precise comparisons of secretome profiles under varying conditions. These protein-based techniques are crucial for discovering biomarkers, understanding disease mechanisms, and developing new therapeutic strategies. In the following, we will discuss some of these methods, including protein microarray, bead-based array, and mass spectrometry.

### 3.1. Methods to Collect Secretome and EVs from Senescent Cells

For the analysis of proteins in the secretome, the following process and related notes should be considered. First, cells should be cultured to the desired confluence and treated to induce senescence. Various methods, including chemical agents or radiation, can be used for this step. Subsequently, cells should be extensively washed with PBS and plated into a chemically defined serum-free culture medium to prevent contamination by extraneous proteins. This should be carried out for overnight (ON) incubation. Afterwards, conditioned media (CM) containing the secretome should be collected and filtered. The samples should then be centrifuged at high speed, and the pellet discarded. This procedure must be performed to eliminate cellular debris and apoptotic bodies [15]. The collected CM containing the secretome of senescent cells can be analyzed through different methods that will be discussed in the following paragraphs.

In addition, the isolation methodology is essential for evaluating the extracellular vesicle (EV) component of the SASP. Considering the role that EVs, such as exosomes and microvesicles, may play in senescence, several protocols using ultracentrifugation and column separations have been reported to specifically isolate EVs from the whole secretome. These purified particles, which contain proteins or nucleic acids, can be used in subsequent experiments analyzing their role in the SASP [9].

### 3.2. Protein Microarray

High-throughput scientific techniques have been created in the last ten years to enhance the investigation of various molecules, such as DNA, proteins, and metabolites. Primarily, DNA microarrays have been used to study the large-scale sequence mutation and deletion identification, transcription factor binding site localization, and gene expression arrays. Nevertheless, DNA microarrays only reveal information on the genes themselves; less information about the produced protein’s role could be obtained. Currently, a new method based on DNA microarray has been suggested, which is called protein microarray [35]. Nowadays, there are three versions of protein microarrays: analytical microarrays, functional microarrays, and reverse-phase microarrays.

Among them, the analytical microarray is normally used to screen a complex mixture of proteins with the aim of affinity binding measurement, specificities, and expression levels of the proteins in the mixture. In this technique, a library of antibodies is necessary to be arrayed on a microscope slide. However, the functional protein microarray is compiled of various arrays which contain the complete sequence of proteins. This method is proper for the investigation of the entire proteome, and or secretome, as it has been used to analyze several protein–protein, protein–DNA, and protein–RNA interactions.

Reverse phase protein microarray (RPA) is the third class of protein microarrays that are associated with analytical microarrays. Cells from different target tissues are separated and lysed for RPA. With the use of a contact pin microarrayer, the lysate is arranged onto a nitrocellulose slide. After that, the slides are probed with antibodies directed against the desired target protein. Chemiluminescent, fluorescent, or colorimetric assays are commonly used to identify the antibodies. To enable protein measurement of the sample lysates, reference peptides should be printed on the slides. The positive point of using an antibody microarray is that this approach can obtain and analyze various types of data in a single experiment. To address some of these characteristics, the study of the variations in the abundance of protein, structural differences in the protein following the modifications or protein isoforms, and the investigation of protein biochemistry could be mentioned. Nowadays, this technique is commercially available thanks to Sigma, Creative biolabs, and Somalogic companies. Generally, in these kits, antibodies are already printed on slides against specific known secretory proteins and the related signal would be detected by fluorescence or chemiluminescence [36].

According to previous studies that have reported the use of antibody microarray for analysis of the secretome, the work of Zhong could be mentioned. In this study, the identification of secretory proteins was accomplished using antibody arrays as a supplementary technique to mass spectrometry. Proteins produced in small quantities during human adipogenesis were identified using adipokine arrays [37].

### 3.3. Bead-Based Array

For targeted protein identification and quantification, antibody and bead-based arrays have been used frequently. In contrast to protein microarrays, in bead-based arrays, the color-coded beads are used to immobilize antibodies. In the next step, fluorescence is used for detection, after which the sample is incubated on the arrays. Furthermore, the identification of multiplexed secretory proteins and bead-based arrays have been utilized. Secretory proteins were diagnosed in cardiomyocytes and fibroblast cells using the bead assay [38]. In 2021, Raj et al. used the bead-based approach to identify the growth factors and cytokines in Mesenchymal stem cells derived from dental pulp, and accordingly, assessed their influence on oral cancer cell development [39]. Furthermore, the miniaturized chip-based systems, Western blotting methods, and ELISA might be used to evaluate the numerous protein expressions in a short time. Recently, a new approach based on combining colocalized-by-linkage assays on microparticles (CLAMP) and bead-based flow cytometry assay has been introduced and named NELISA. The CLAMP technology is a new sandwich assay that uses pre-immobilizing antibody pairs on the surface of microparticles and has been claimed to be preventive for the formation of reagent-driven cross reactivity. The detection of aimed proteins is based on the displacement approach. The NELISA method has been used to detect the growth factors, chemokines, and low-abundance cytokines, and the positive point of this method is about fast rate processing. As mentioned by the authors, it is possible to identify 191 proteins from 7392 samples in less than 7 days [40].

These protein chip-based techniques have several benefits and drawbacks. The positive points of using protein microarrays are as follows: (1) The method is sensitive for identifying proteins with low abundance. (2) It is widely reproducible and a highly specialized procedure. Subsequently, to address the weaknesses of this method, the following issues might be considered: (1) One of the major barriers is the cost of this method in the case of specific antibodies, since this method relies on specific and high-affinity antibodies available for both capture and detection. (2) Non-specific binding of the antibodies to other proteins is possible. (3) Rapid denaturation, or disulfide bond breakage, is a risk factor for several secretory proteins that can impact binding.

### 3.4. Mass Spectrometry

Proteomics techniques have been used for two crucial types of evaluations: top-down secretomics and bottom-up secretomics. In general, these two distinct methods are used in various instruments, for instance, mass spectrometers with various ionization sources. The preferred mass spectrometer in top-down secretomics is referred to as MALDI-MS, and in bottom-up secretomics, the preferred mass spectrometer is known as ESI-MS, with an electrospray ionization source.

In the top-down secretomics approach, there is no need for prior digestion or fractionation. This method analyses the secretome or secreted protein profiles both for low-molecular and high-molecular mass by using the MALDI-MS. The point is that after detecting the variations between samples, the SDS-PAGE technique is required for further analysis. One of the most applicable techniques that could be considered in this step is the two-dimensional electrophoresis. In this technique, proteins are first separated by mass (SDS-Polyacrylamide gel electrophoresis, SDS-PAGE) and then by net charge (isoelectric focusing, IEF) in the second dimension [41]. Accordingly, to identify the proteins, they should be resolved as distinct spots and analyzed by mass spectrometry [42].

To address one of the drawbacks of the top-down secretomics approach, it might be mentioned that this method is not successful in the case of posttranslational modified proteins that are a considerable number of secreted proteins in eukaryotic cells [43]. Consequently, the observed peak in the MALDI-MS spectrum could not be related to the target protein, but nonetheless, might signify a protein which has been modified by posttranslational process [44,45]. Even though observed changes are not at the naked protein level, they might even be revealed, and the related protein will be identified in this way. As it is reported by Champion et al. for the investigation of Mycobacterium marinum by using MALDI–time-of-flight (TOF)–MS for the single colony, ESAT-6 and CFP-10 which are the major substrates of type VII secretion of ESX-1, have been identified [46].

In bottom-up secretomics, an enzyme, such as trypsin, is used to digest the secretome and then LC-MS/MS is used for the result evaluation [47,48]. Alteration between samples will be determined either before two-dimensional SDS-PAGE or after protein digestion. As the proteins containing posttranslational modification will be digested into different portions, the non-modified portions could be investigated by using LC-MS/MS. This not only might lead to peptide sequence determination, but also could lead to the identification and quantification of the protein. To mention the positive points of this approach, sensitivity, and reliability for providing sequence information could be considered. Nevertheless, during the analysis, degraded proteins are not being considered [45,49,50]. Thus, if a quantitation is carried out, the findings will be inaccurate or falsely positive. To overcome this issue, it is necessary to employ two or three peptides for quantification in order to quantitate the objects [51,52,53].

In this field, there are many studies that have been carried out by using mainly two different methods in the first step of their investigation as follows; first; culturing either unstimulated or stimulated cell cultures for one to two days, then collecting, concentrating in the case of a low amount of proteins in the supernatant, fractionating the mixture, and finally using mass spectrometry to identify the released proteins. The second method is an alternative one which considers body fluids as a substitute for conditioned media. In 2012, Stastna and Van Eyk used the approach of using conditional media to find secreted proteins that would be suitable candidates for biomarkers in cardiovascular disorders [54]. However, the same approach has been used by Helena Skalnikova to find proteins involved in cell survival, proliferation, differentiation, or inflammatory response; this method was also used for secretome profiling of primary isolates of astrocytes, endothelial cells, muscle cells, and mesenchymal, adipose, neural, and embryonic stem cells [55].

As mentioned above, the previous approaches that have been used were dependent on protein electrophoresis. In fact, in those methods, the protein samples should first be proceeding the electrophoresis analysis, either 2-DE or difference gel electrophoresis (DIGE). In 2-DE, proteins will be separated into two different levels, first based on their net charge, and subsequently based on their size. However, in the DIGE approach, proteins should be labeled with various fluorescent dyes, including Cy2, Cy3, and Cys5 prior to electrophoresis [56]. Accordingly, both separation processes (net charge and size) will be performed in a single gel. In the final step, the mentioned gel will be screened by using fluorescence imaging.

There are also some techniques based on spectrometry which do not depend on electrophoresis, for instance, isotope-coded affinity tag (ICAT), isobaric tag for relative and absolute quantization (iTRAQ), stable isotope labeling by amino acids in cell culture (SILAC), selected reaction monitoring (SRM), and surface-enhanced laser desorption/ionization time-of-flight mass spectrometry SELDI-TOF MS. These approaches are considered as quantitative mass spectrometry techniques which are capable of making a direct comparison of protein levels in different samples. So, they are reliable methods which give us inclusive data about cell protein secretion in different stages.

In the ICAT method, light and heavy ICAT reagents are used to label two different protein samples by being bonded to the cysteine residue. Since these reagents contain biotin tag, the mixed and digested samples will be regained by using affinity chromatography followed by LC–MS/MS [57]. This approach has been used for the analysis of secretome of human glioma cells and their tumor microenvironment [58]. The other version of this approach, which is called cleavable ICAT (cICAT), uses an acid-cleavable linker and 12C or 13C isotopes [59]. This method has been employed to study the secretome profile of non-transformed human epithelial cell line under conditioned media. There have been 125 novel proteins found, 49 of which have been found to be strongly down-regulated by c-Myc, which indicates a notable enrichment of components linked to cellular senescence and growth suppression [60]. In another study, the analysis of adipocytes by this approach has led to the identification of 77 reported adipokines, such as adiponectin, cathepsin D, cystatin C, resistin, and transferrin [61]. Moreover, 240 proteins released by adipocytes have been discovered; most of these proteins showed increased expression in response to insulin treatment.

However, the iTRAQ method allows for the comparison of the relative abundance of proteins across four samples in a single mass spectrometric experiment. Four tag reagents that attach to the peptide’s N-terminus are used by iTRAQ. Therefore, the main amines of peptides and proteins are labeled by iTRAQ. Following independent digestion and mixing of each sample with the appropriate iTRAQ tag, LC-MS/MS will be performed [62]. This technique has been used to study the secretory proteins related to the immune system of crayfish hemocytes before and after infection by Spiroplasma eriocheiris [63]. Moreover, the use of iTRAQ for analysis of the testicular interstitial fluid secretome related to aging and senescence in aged mice has been reported [64].

Stable isotope labeling by amino acids in cell culture (SILAC) is the technique in which light and heavy isotopes of essential amino acids are used to mark cell culture medium from two samples that are deficient in amino acids. The essential amino acids arginine and/or lysine, which cannot be synthesized by mammalian cells, are labeled carbon-13, nitrogen-15, or deuterium isotopes [65]. In the next step, after digestion by trypsin, samples are used for liquid chromatography mass spectrometry [66]. This approach is one of the most applicable methods for mammalian and plant cells. Furthermore, the application of this method for a variety of cancer cells secretome analysis, including gastric cancer [67], breast cancer [68], and lung cancer [69], has been reported.

Selective reaction monitoring (SRM), or multiple reaction monitoring (MRM), is a label-free method for accurately quantifying low-abundance analytes in complicated mixtures. Triple-quadrupole (QQQ) mass spectrometers are commonly used for the SRM approach. Quadruple Q1 isolates the peptide precursor ion, Q2 functions as a collision cell where the precursor is fragmented, and Q3 is used to identify a particular fragment ion. In spite of the other MS methods, SRM analysis does not record entire mass spectra. The precise identification of low-abundance proteins in extremely complicated samples is ensured by selecting and detecting a single pair of precursor and fragment ions rather than scanning the whole mass spectra. This results in great selectivity, sensitivity, and a dynamic range spanning up to five orders of magnitude [70]. SRM quantification is a useful technique for screening more samples, and in some circumstances, it may be used in place of Western blotting to confirm the findings of proteome studies.

### 3.5. SELDI-TOF Mass Spectrometry

Hutchens and Yip initially described surface-enhanced laser desorption/ionization time-of-flight mass spectrometry (SELDI-TOF MS) as a proteomic method in 1993 [71]. This is a high-throughput technique that involves protein separation and analysis after attaching a crude sample to a protein chip array. This technique is very sensitive to proteins and peptides with molecular weights less than 15 kDa [72]. The advantage is that this approach does not require previous protein separation, so classified as gel-independent techniques for protein analysis. SELDI-TOF MS could be employed to study several types of molecules, such as nucleic acids, organic molecule solutions, proteins, and even bacteria [73]. As an advantage of this approach, the possibility of using even a small number of samples could be mentioned [24].

In 2006, in a study of secretome for embryonic by using SELDI-TOF MS, individual secretome signatures were observed at various times, which led to the finding of some embryonic stage-specific proteins [74]. In the other study, this high-throughput method has been applied to study the secreted proteins between Hepatocellular Carcinoma and adjacent normal liver tissue [75]. In Table 2, a summary of information about all the methods related to investigate the proteins in the secretome of cells has been prepared considering their main features, advantages, and limitations.

## 4. Secretome Analysis Methods Based on Nucleic Acid

Secretome analysis methods based on nucleic acid involve techniques to study the collection of proteins secreted by cells, using nucleic acid tools to identify and quantify these proteins. These methods can include RNA sequencing to infer protein secretion, DNA microarrays to detect gene expression linked to secreted proteins, and various nucleic acid amplification techniques to enhance sensitivity. Such approaches are crucial for understanding cell signaling, disease mechanisms, and developing therapeutic strategies. In the following, RNA sequencing, DNA microarray, and serial analysis of gene expression (SAGE) will be discussed.

### 4.1. RNA Sequencing

Currently, high-throughput techniques of RNA sequencing could be used to investigate and analyze the transcriptome profile. In this technique, a complementary DNA (cDNA) ought to be produced through reverse transcriptase assay from the total mRNA of the organism. Accordingly, the cDNA will be fragmented and sequenced using various technologies, such as Roche454, Illumina, and Solid, to generate the transcriptome map. One of the advantages of this method is that the background noises are considerably lower than DNA microarray. The single-base resolution version could be used in the case of low RNA molecules, as it requires a small amount of RNA molecule to perform the analysis. When it comes to RNA sequencing applications, quantifying the mRNA abundance, performing transcription mapping, splicing patterns, and gene expression analysis could be considered. One of the applications of this approach is for unraveling the secretome profile of particular cells. In this case, after sequence alignment, SignalP could be used in order to anticipate the cleavage sites, as well as signal peptide tags.

Like all the techniques, RNA sequencing also has advantages and weaknesses for the secretome analysis. To address the advantages of this approach, the following points could be considered: first, there is no need for any previous information on genome sequence; the second point is that any organism could be applied in contrast to the approaches that background information of model organisms is necessary.

Nonetheless, there are some disadvantages to using RNA sequencing for secretome studies. One of the most reported disadvantages is that a vast majority of sequences will be produced, which subsequently need to undergo a process in which secretory proteins would be predicted. Since there would be a high amount of data, storing the data and analysis could be challenging and time consuming. Furthermore, due to the fact that this technique is a type of structural test, the only thing that would be obtained is the sequence information. Even though in single-base resolution RNA sequencing it is possible to conduct the experiment with a lower amount of RNA sample, in the case of rare transcripts, this technique might not be efficient in representing the related data.

### 4.2. DNA Microarray

DNA microarray is generally used for measuring the mRNA concentration or, in other words, gene expression investigation by using a DNA probe complementary to the mRNA of the interested gene. In some cases, it is possible to use different DNA probes matching all the mRNAs, which leads to having an expression profile of total genes expressed in a particular moment. There are two types of microarrays: cDNA arrays (spotted array) and oligonucleotide arrays (GeneChips). High-density probes with a length of about 25 nucleotides are generated directly on the chip in oligonucleotide arrays. These arrays are coated with labeled RNA molecules. Hybridization results in the generation of a fluorescent signal. These arrays are used to quantify gene expressions using a single-color detection technique. Glass slides are spotted with cDNA probes that have been generated for the spotted array. The array is hybridized with RNA molecules that have been tagged with Cy3 and Cy5. Gene expression monitors by using a two-color detection approach.

This technique has been used in different studies for the investigation of the secretome of tissues and cells. In 2012, Hoggard et al. compared the secretome of human omental with subcutaneous adipose tissue by applying DNA microarray [76]. Additionally, in another study, it was reported that secretome was analyzed by using DNA microarray in human fat, which led to the discovery of eight adipogenesis-related adipokines [77]. This approach has also been used to study the cancer cell secretome. As an example, Dombkowski and his group were working on breast cancer cells based on gene expression profiles obtained from DNA microarray; they reported two clusters of secretome genes related to proliferative breast disease progression [78].

DNA microarray is cost effective concerning RNA sequencing; however, mRNA expression does not always correlate with the related protein expression and could be considered. Thus, it would be complicated to obtain the actual secretome in a particular given condition.

### 4.3. Serial Analysis of Gene Expression (SAGE)

Serial analysis of gene expression is a diagnosis method based on the tag with a length of about 10 bp that can measure the total gene expression pattern [79]. This method was developed in 1995 by Velculescu at Johns Hopkins University. In fact, this assay is a sequence-based approach that could diagnose which gene is expressing and could measure the expression level of that in a particular time. In this approach, after RNA isolation, complementary DNA will be produced by using biotinylated oligo primers. Subsequently, this cDNA will bind to Streptavidin beads after being digested by an anchoring enzyme. Diagnosis will be performed according to the ligation of the designed tags to the reaction mixture. This technique could be a proper choice for the analysis of secretome of cells since despite the other methods, SAGE assay could be performed for both quantitative and qualitative analysis for the unknown transcripts. There are a variety of studies in which this method has been used for human, animal, and yeast gene expression investigation and several modifications of this assay have been invented, including, MAGE, SADE, deepSAGE, microSAGE, miniSAGE, longSAGE, and superSAGE [80]. In Table 3, an overview of secretome analysis approaches based on nucleic acids has been provided, highlighting their descriptions, advantages, disadvantages, and applications.

## 5. Conclusions

The exploration of the secretome, defined as the complex array of proteins released by cells under various conditions, has significantly advanced our understanding of cellular processes, disease mechanisms, and potential therapeutic targets. Currently, secretome analysis has emerged as a powerful tool, offering insights into immunological responses, development, cellular senescence, and the pathogenesis of diseases such as cancer. Through diverse methods, including in silico prediction, nucleic acid-based techniques such as RNA sequencing and DNA microarrays, and protein-based approaches like protein microarrays and mass spectrometry, researchers have uncovered the intricate composition and dynamics of the secretome.

SecretomeP and SignalP are two examples of bioinformatics tools that might be used for in silico research to predict secreted proteins rapidly and economically. However, because it depends on signal peptides, it cannot identify non-classical secretory proteins as well; for this reason, supplementary experimental validation is necessary. RNA sequencing and DNA microarrays are considered nucleic acid-based techniques that provide details on the transcriptome landscape of secretome profiles and make it possible to quantify the levels of messenger RNA and gene expression patterns. DNA microarrays, on the other hand, enable affordable gene expression monitoring, whereas RNA sequencing offers single-base precision and better application across species.

Comprehensive protein-level studies of secreted proteins are achievable with protein-based methods like mass spectrometry and protein microarrays. While mass spectrometry allows both top-down and bottom-up techniques for protein identification and quantification, protein microarrays provide high-throughput screening and functional investigation of secretome components. Understanding the involvement of secreted proteins in a range of biological processes and disorders, from immunomodulation and tissue repair to the advancement of cancer and aging, has been proven conceivable mainly in large part due to these methodologies.

The complicated interactions between disease states and secreted substances are highlighted by the study of cellular senescence. Senescent cells are linked to cancer defense systems and aging. They are distinguished by halted cell division and the SASP factors production. The significance of secretome analysis in biomedical research is demonstrated by an understanding of the SASP and its implications for tissue homeostasis and pathogenesis.

The study of the secretome endures several obstacles in spite of tremendous progress, such as the requirement for improved approaches to identifying non-classical secretory proteins, resolving the deficiencies of current methodologies, and incorporating multi-omics data for extensive evaluation. Further research ought to concentrate on enhancing experimental methodologies, expanding our understanding of secretome dynamics in both health and disease, and utilizing this information for therapeutic purposes, like biomarker identification and targeted therapies.

To conclude, the secretome represents a frontier in biomedical research that will allow us to gain insight into the intricacies of disease mechanisms, cellular communication, and treatments. Progress in this field will be fostered by ongoing multidisciplinary cooperation and technological advancements, which will eventually improve human health and quality of life.

## Figures and Tables

**Table 1:** Overview of In Silico Tools for Secretome Analysis.

Tool	Description	Advantages	Limitations
SecretomeP	Predicts non-classical secretory proteins	Identifies non-signal peptide proteins	Risk of false predictions
SignalP	Predicts proteins with signal peptides	Well established, reliable for classical pathways	Missing non-classical secretory proteins

**Table 2:** Protein based methods, main features, advantages, and disadvantages.

Methods	Features	Advantages	Disadvantages
Protein microarrays	- Analytical, functional, and reverse-phase microarrays - Uses antibodies arrayed on microscope slides - Identifies protein-protein, protein-DNA, and protein-RNA interactions - Detects proteins via chemiluminescent, fluorescent, or colorimetric assays	- Sensitive to low-abundance proteins - High reproducibility - Commercially available kits - Simultaneous analysis of multiple proteins in a single experiment	- High cost of specific antibodies - Non-specific antibody binding - Risk of protein denaturation or disulfide bond breakage
Bead-based array	- Uses color-coded beads to immobilize antibodies - Detection via fluorescence - Can be used for multiplexed secretory protein identification - NELISA combines CLAMP and bead-based flow cytometry assays for rapid processing	- Suitable for high-throughput and multiplexed assays - Fast processing rate - Prevents reagent-driven cross-reactivity	- Requires specialized equipment - Potential for non-specific binding - Can be complex to set up and optimize
Mass spectrometry	- Top-down and bottom-up secretomics - MALDI-MS for top-down, ESI-MS for bottom-up - Top-down analyzes intact proteins - Bottom-up uses enzyme digestion and LC-MS/MS	- Sensitive and reliable for sequence information - Capable of identifying post-translational modifications - Can analyze a wide range of proteins from different sources	- Top-down not effective for post-translationally modified proteins - Bottom-up may miss degraded proteins - Quantitation can be inaccurate if only one peptide is used
ICAT	- Labels proteins with light and heavy ICAT reagents bonded to cysteine - Uses affinity chromatography and LC-MS/MS - Can analyze secretomes of various cell types and conditions	- Allows for comparison of protein levels across different samples - Effective for identifying post-translational modifications	- Limited to proteins with cysteine residues - Requires specialized reagents and equipment
iTRAQ	- Labels peptides at the N-terminus with four tag reagents - Allows comparison across four samples in a single experiment - Uses LC-MS/MS for analysis	- Enables relative quantification of proteins across multiple samples - High sensitivity and specificity	- Can be expensive - Requires extensive sample preparation and optimization
SILAC	- Uses light and heavy isotopes of essential amino acids to label cell culture medium - Analyzes samples via liquid chromatography mass spectrometry (LC-MS)	- Effective for analyzing mammalian and plant cells - Suitable for cancer cell secretome analysis - Provides accurate quantification of protein expression	- Limited to cell cultures that can incorporate labeled amino acids - Requires specialized isotopes and equipment
SRM/MRM	- Label-free quantification of low-abundance analytes - Uses triple-quadrupole mass spectrometers - Does not record entire mass spectra	- High selectivity, sensitivity, and dynamic range - Suitable for screening numerous samples - Can replace Western blotting for confirming proteome study findings	- Requires specialized equipment and expertise - May not be suitable for all types of samples
SELDI_TOF MS	- High-throughput technique - Protein separation and analysis without prior electrophoresis - Sensitive to proteins and peptides with molecular weights less than 15 kDa	- Does not require previous protein separation - Can be used with small sample quantities - Applicable to a wide range of molecules including proteins, nucleic acids, and bacteria	- Limited to low-molecular-weight proteins - May require extensive optimization for different sample types

**Table 3:** Overview of secretome analysis approaches based on nucleic acids.

Method	Description	Advantages	Disadvantages	Applications
RNA Sequencing	Analyzes transcriptome by sequencing cDNA produced from mRNA	No need for prior genome info; less background noise	Large data volume; only sequence info; less efficient for rare transcripts	mRNA quantification, gene expression analysis, splicing patterns
DNA Microarray	Measures mRNA concentration using complementary DNA probes	Cost effective; can profile total gene expression	mRNA may not correlate with protein expression	Tissue and cell secretome investigation, gene expression profiling
SAGE	Uses short tags to measure gene expression patterns	Quantitative and qualitative analysis; applicable to various organisms	Requires specific enzymatic reactions	Diagnosing gene expression, measuring expression levels over time

## Data Availability

Not applicable.
